# Spontaneous Osteomyelitis and Intraosseous Abscess: A Case Report

**DOI:** 10.5811/cpcem.6568

**Published:** 2024-06-03

**Authors:** Meghan Chamberlain, Simon A. Sarkisian

**Affiliations:** Cooper University Hospital, Department of Emergency Medicine, Camden, New Jersey

**Keywords:** *osteomyelitis*, *intraosseous abscess*, *pediatric*, *case report*

## Abstract

**Introduction:**

Acute hematogenous osteomyelitis may have significant morbidity and mortality if undiagnosed. Because it is uncommon in developed countries and has variable presentations, the patient may undergo several healthcare visits prior to diagnosis.

**Case Report:**

We report the case of a 9-year-old male who presented with hip and knee pain with associated fevers and was found to have osteomyelitis and intraosseous abscess in the diaphysis of the right femur. He had multiple emergency department and outpatient visits before the ultimate diagnosis was made. He was treated with irrigation and debridement in addition to intravenous antibiotics.

**Conclusion:**

Pediatric acute hematogenous osteomyelitis can have subtle presentations, and this case illustrates some of the difficulties in making the diagnosis. This condition should be considered in the workup of a child with undifferentiated fever, pain, or decrease in mobility.

Population Health Research Capsule What do we already know about this clinical entity?

*Acute hematogenous osteomyelitis is well characterized in the literature with regard to the physiology, epidemiology, presentation, and management.*

What makes this presentation of disease reportable?

*Aside from the reported history, this pediatric patient had a clinically benign presentation of this uncommon disease process.*

What is the major learning point?

*Consider diagnosis of acute hematogenous osteomyelitis in patients with localized pain and recurrent fever, even if the physical exam is underwhelming.*

How might this improve emergency medicine practice?

*Clinicians should consider this diagnosis more frequently and consider more intensive evaluation.*

## INTRODUCTION

Acute hematogenous osteomyelitis is caused by bacteria proliferating within the bone. It occurs from hematogenous spread, where episodes of bacteremia combine with turbulent flow at the metaphyseal arteriolar-venous sinusoid transition to allow local invasion. These episodes occur more commonly during childhood, as the immature bone is less capable of containing infection. Most cases involve children younger than five years of age.^1^ Local trauma contributes to risk of hematogenous infection, presumed to be secondary to microvascular bleeding and stasis. After age one, male children are much more likely to sustain minor trauma than female children, which may explain why osteomyelitis disproportionately affects males.^2^ Other causes include direct inoculation from a procedure or trauma such as intraosseous access or spread from surrounding tissues.

In older patients, the bones are thicker and the periostea is denser; so osteomyelitis is more likely to cause formation of an intraosseous abscess. Infections are predominantly caused by *Staphylococcus aureus*, with about one third being methicillin resistant.^3^ Patients with sickle hemoglobinopathies are at specific risk for infection from salmonella.^4^ Patients can have a variety of presentations including fevers, irritability, decreased extremity function, point tenderness, swelling, and warmth. Because *S aureus* has variable growth and the inoculum may be small, the clinical course may be indolent and patients may present without fever or with only low-grade fever. Risk factors include sickle cell disease or any immunocompromised state, sepsis, minor trauma, and indwelling vascular catheters.^5^

## CASE REPORT

A nine-year-old male with history of Hashimoto thyroiditis and Raynaud disease presented to the emergency department (ED) for intermittent right hip and knee pain, daily fevers, and difficulty with ambulation for 16 days. He had no prior history of injury to the leg or of intraosseous access. He had initially presented to a different ED on day 10 of illness where he had a normal hip radiograph and labs. The patient was evaluated for autoimmune and tick-borne illnesses. He was then referred to an infectious disease specialist whom he saw on day 16. The patient was ultimately sent to our ED for further evaluation and imaging.

**Image 1.**
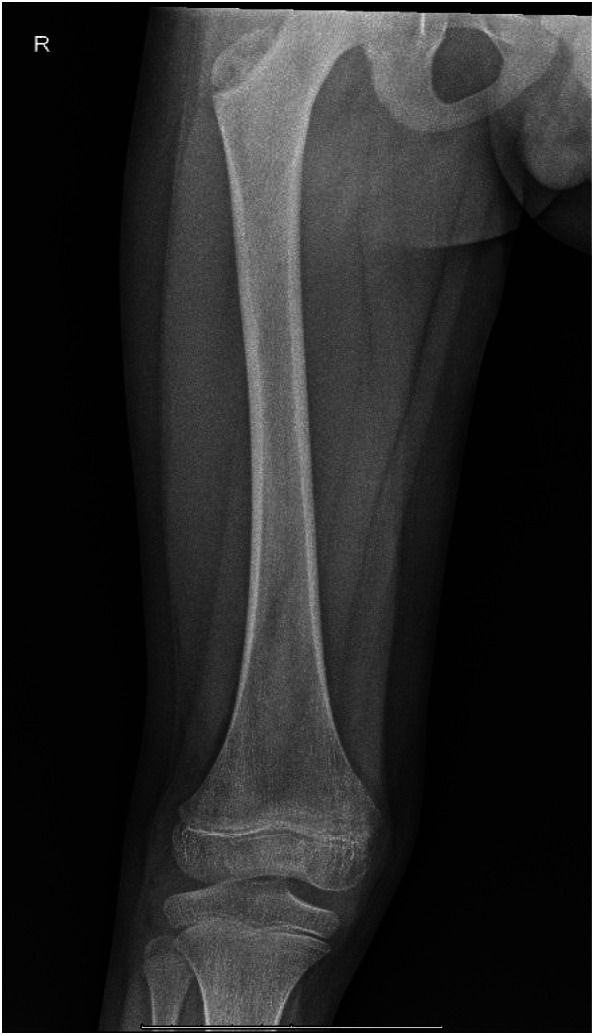
Radiograph of the right femur upon emergency department presentation with no acute abnormalities.

Vital signs were notable for a temperature of 37.9° Celsius orally and heart rate of 155 beats per minute. On physical exam, the patient had no tenderness to palpation of the right lower extremity, normal range of motion of all joints, and was able to ambulate and jump without pain. No swelling of the hip or overlying skin changes were noted. The patient’s laboratory testing was notable for C-reactive protein (CRP) elevated at 16.82 milligrams per deciliter (mg/dL) (reference range 0.3–1.0 mg/dL), erythrocyte sedimentation rate (ESR) above assay limits at greater than 100 millimeters per hour (mm/hr) (l0–15 mm/hr in males, 0–20 mm/hr in females), but no leukocytosis. Radiographs of his right hip, right knee, and right femur were unremarkable (Image 1).

**Image 2.**
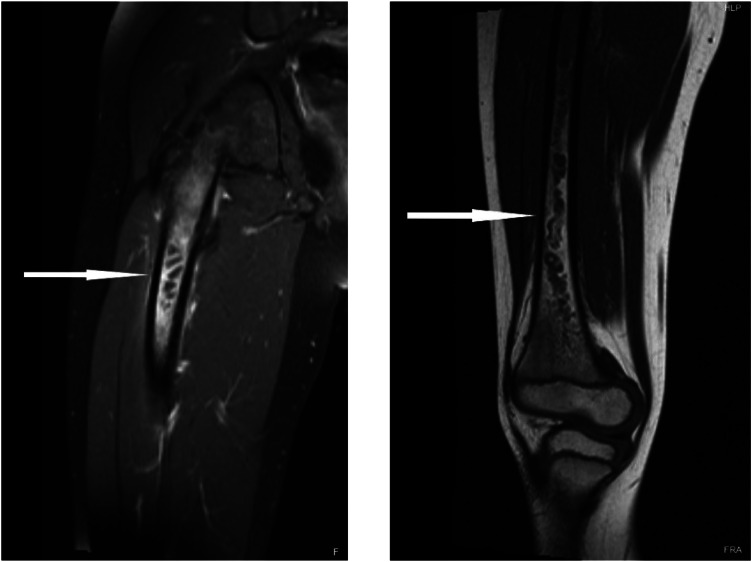
Magnetic resonance imaging of the right femur with contrast demonstrating lesions in the proximal (left) and mid (right) femoral diaphysis (arrows).

The patient was admitted for further evaluation and found to have continued intermittent fevers and right lower extremity pain. On Day 17, he had magnetic resonance imaging (MRI) of the right lower extremity with and without contrast, which revealed right femoral osteomyelitis with underlying intraosseous abscess (Image 2). He was taken to the operating room for a right femur reaming and irrigation with debridement. Blood and tissue cultures grew methicillin susceptible *S aureus*, and workup was notable for an echocardiogram without evidence of vegetation and abdominal ultrasound without evidence of an intra-abdominal source of infection. He received intravenous cefazolin for 30 days and was then transitioned to two weeks of oral antibiotics, starting with doxycycline, which was then changed to oral levofloxacin due to side effects. His inflammatory markers were trended throughout therapy until they normalized. Patient had resolution of his symptoms and was able to ambulate without abnormality.

## DISCUSSION

Acute hematogenous osteomyelitis is uncommon, with an estimated incidence of eight per 100,000 children per year.^3^ Diagnosis can be subtle, and patients often present with pain that is not well localized and without signs of local infection. Patients typically present within one week of onset, but diagnosis can require multiple visits, as occurred with our case.^6^

A systemic review of the literature by Darnell et al found that presenting symptoms were most commonly pain (81.1%), localized signs or symptoms of infection, such as warmth, point tenderness, or swelling (70%), fever (61.7%), reduced range of motion (50.3%), and reduced weight-bearing (49.3%). The most common laboratory abnormalities were elevated inflammatory markers (ESR 91%, CRP 80.5%), with or without leukocytosis (35.9%). They also found that initial imaging was limited with sensitivity of radiograph 16-20%, computed tomography (CT) 67%, ultrasound 55%, while other imaging modalities had significantly better sensitivity with bone scan 53–100% and MRI 80–100%. The decision to perform MRI with contrast in children is controversial as there is no evidence that contrast improves sensitivity or specificity of detection.^7^ Because MRI requires deep sedation or general anesthesia in young children, children with concerning clinical or lab findings are usually hospitalized for close monitoring and/or treatment pending MRI imaging.

In a systematic review examining intraosseous abscess (termed Brodie abscess) by Van der Naald et al, 407 patients were examined. Of these, the median time to diagnosis from initial complaint was 12 weeks with primary presenting complaints of pain (98%) and swelling (53%). Patients typically had full resolution of symptoms; however, two of the included patients (0.5%) had lasting sequelae from bony destruction: limb shortening, and vertebral body collapse complicated by neurologic deficits.^3^

This case demonstrates the difficulties in evaluating for this condition. While the patient’s history was concerning for possible infection, upon presentation to the ED he did not have any localizing exam findings to suggest underlying infection and had no identifiable risk factors for development of this condition. This case was uniquely difficult as well given that the abscesses were localized to the diaphysis of the bone, whereas lesions are typically located in the metaphysis or epiphysis. Further complicating the diagnosis was the patient’s history of autoimmune conditions, namely Hashimoto thyroiditis and Raynaud syndrome, which suggested a possible autoimmune etiology of his symptoms. There is no established relationship between the development of osteomyelitis and these conditions. The differential for this presentation is extensive and includes tick-borne illnesses, autoimmune conditions, musculoskeletal injuries, and infection.

## CONCLUSION

Acute hematogenous osteomyelitis can have serious consequences if undiagnosed, including sepsis and bony destruction. Not all patients will have identifiable risk factors and presentations may be subtle. Many patients will lack focal tenderness or swelling on exam, and the diagnosis should be considered in patients who have recurrent fevers or complain of localized pain.
